# Supplementary material for: Pregnant women’s attitudes towards complementary and alternative medicine and the use of phytotherapy during the COVID-19 pandemic: A cross-sectional study
Source: PLoS One. 2024 Jan 2;19(1):e0296435. doi: 10.1371/journal.pone.0296435 (PMC10760753; doi:10.1371/journal.pone.0296435)
Supplement: S2 Table — (DOCX) [file pone.0296435.s002.docx]

**Table.** **Herbal products used by pregnant women (n=381)**

| **Herbal name** | | **Users** | | **Those who do not use** | |
| --- | --- | --- | --- | --- | --- |
|  |  | **n** | **%** | **n** | **%** |
| Ginger | *Zingiber officinale* | 131 | 26.0 | 373 | 74.0 |
| Turmeric | *Curcuma longa* | 137 | 27.2 | 367 | 72.8 |
| Fennel | *Foeniculum vulgare* | 98 | 19.4 | 406 | 80.6 |
| Daisy | [*Matricaria chamomilla*](https://www.google.com.tr/search?sxsrf=APq-WBvlEPQK3da5euEzQ9DyLmjeNxAJgw:1648421173084&q=Papatyan%C4%B1n+Latince+ismi+nedir%3F&sa=X&ved=2ahUKEwib1Nizr-f2AhW_R_EDHdLjCzAQsZYEegQIDxAC) | 86 | 17.1 | 418 | 82.9 |
| Mint and lemon | *Mentha piperita-Citrus limonum* | 188 | 37.3 | 316 | 62.7 |
| Rosehip | *Rosa canina* | 94 | 18.7 | 410 | 81.3 |
| Echinacea | *Echinacea purpurea* | 57 | 11.3 | 447 | 88.7 |
| Cumin | *Cuminum cyminum* | 147 | 29.2 | 357 | 70.8 |
| Green tea | *Camellia sinensis* | 74 | 14.7 | 430 | 85.3 |
| Sage tea | *Salvia officinalis* | 67 | 13.3 | 437 | 86.7 |
| Raspberry | *Rubus idaeus* | 36 | 7.1 | 468 | 92.9 |
| Aloe vera | *Tıbbi sarısabır* | 21 | 4.2 | 483 | 95.8 |
| Lion claw | *Allcemilla vulgaris* | 18 | 3.6 | 486 | 96.4 |
| Avocado | *Persea americana* | 72 | 14.3 | 432 | 85.7 |
| broad beans | *Vicia faba* | 69 | 13.7 | 435 | 86.3 |
| Okra | *Abelmoschus esculentus* | 96 | 19.0 | 408 | 81.0 |
| Black elderberry | *Sambucus nigra* | 33 | 6.5 | 471 | 93.5 |
| Date | *Phoenix dactylifera* | 47 | 9.3 | 457 | 90.7 |
| midwife | *Malva sylvestris* | 57 | 11.3 | 447 | 88.7 |
| Plum | *Prunus domestica* | 81 | 16.1 | 423 | 83.9 |
| Cardamom | *Elettaria cardamomum* | 42 | 8.3 | 462 | 91.7 |
| Garlic | *Allium sativum* | 249 | 49.4 | 255 | 50.6 |
